# Persistence and Organ Tropism of Filoviruses in Farmed European Perch (*Perca fluviatilis*)

**DOI:** 10.1111/jfd.70105

**Published:** 2025-12-06

**Authors:** Johanna Perschthaler, Nicole Wildi, Lena K. Matthiss, Torsten Seuberlich, Heike Schmidt‐Posthaus

**Affiliations:** ^1^ Department of Infectious Disease and Pathobiology, Vetsuisse Faculty, Institute for Fish and Wildlife Health University of Bern Bern Switzerland; ^2^ Division of Neurological Sciences, Department of Clinical Research and Veterinary Public Health, Vetsuisse Faculty University of Bern Bern Switzerland

**Keywords:** aquaculture, European perch, Filoviridae, systemic infection

## 1 Introduction

The European perch, *Perca fluviatilis* , is a promising fish for freshwater aquaculture (Overton et al. 2015). However, infectious diseases and the limited knowledge of their diagnosis and treatment are critical bottlenecks for effective farming (Overton et al. 2015; Policar et al. 2019). Viral infections, for example, by perch rhabdoviruses, are reported to cause high mortalities in perch aquaculture (Bigarré et al. 2017), but little is known about other viruses infecting European perch and their relevance to animal health.

Filoviruses are negative‐sense single‐stranded RNA viruses belonging to the family Filoviridae in the order Mononegavirales. Prominent examples of filoviruses are Marburg virus and Ebola virus, which cause severe haemorrhagic fever in humans with high lethality and mortality (Kuhn et al. 2020). Until recently, only filoviruses affecting mammalian species (primates, bats and pigs) were known. This changed with the first reports of filoviruses in fish (Huángjiāo virus in captured frogfish and Xīlǎng virus in captured filefish), indicating that the genetic diversity and host range of filoviruses may be much larger than currently known (Shi et al. 2018).

In a previous meta‐transcriptomic virus discovery study, our laboratories identified four novel filoviruses in organ pools of European perch that derived from an aquaculture farm in Switzerland and were purchased as juveniles from a farm in Germany in 2017: FIWI virus (FIWIV), Kander virus (KNDV), Oberland virus (OBLV) and Loetschberg virus (LOEBV) (Table 1) (Hierweger et al. 2021; Seuberlich et al. 2023). However, with the available tissue samples at the time of that study, we could not address questions related to the putative association of the infection with disease and pathogenesis. In 2023, 6 years after the first detection of these filoviruses, European perch from the same facility in Germany were sent to our diagnostic services for a follow‐up examination, allowing us to expand our investigations.

**TABLE 1:** Overview of fish filoviruses, related hosts, and their taxonomic classification by the International Committee on Taxonomy of Viruses.

Virus name	Genus, species	Host	References
Xīlǎng virus	*Striavirus*, *Striavirus antennarii*	*Antennarius striatus*	Shi et al. (2018)
Huángjiāo virus	*Thamnovirus*, *Thamnovirus thamnaconi*	*Thamnaconus septentrionalis*	Shi et al. (2018)
Fiwi virus	*Thamnovirus*, *Thamnovirus percae*	*Perca fluviatilis*	Hierweger et al. (2021)
Kander virus	*Thamnovirus*, *Thamnovirus kanderense*	*Perca fluviatilis*	Hierweger et al. (2021)
Oberland virus	*Oblavirus*, *Oblavirus percae*	*Perca fluviatilis*	Hierweger et al. (2021)
Loetschberg virus	*Loebevirus*, *Loebevirus percae*	*Perca fluviatilis*	Seuberlich et al. (2023)

The aims of this study were: (1) to determine whether filoviruses persisted in the European perch farm of origin, (2) to characterise their organ distribution in infected fish and (3) to investigate whether the presence of the viruses is associated with organ lesions and disease.

## 2 Materials and Methods

Adult European perch (*n* = 32) were sent to the Institute for Fisch and Wildlife Health, University of Bern from an aquaculture farm in Saxony, Germany, for a diagnostic follow‐up of a previous disease event (Hierweger et al. 2021). The animals were kept in the same earth pond as the previous batch and showed no clinical signs. They were killed on‐site and shipped at 4°C. Upon arrival at the laboratory, the total weight and length of individual animals were recorded, followed by a full necropsy. The condition index of all fish was calculated according to Fulton's *K* condition index (Heincke 1908):

$$K=100\times \frac{\text{weight}}{{\text{length}}^{3}}$$

External and internal organ lesions were assessed. Tissue samples of brain, liver, spleen, kidney, gills and pancreas including pieces of fatty tissue and intestine were collected, transferred to 1.5 mL Eppendorf tubes, snap frozen in liquid nitrogen and stored at −80°C until further analysis. Total RNA was extracted from tissue samples using TRI Reagent (Merck, Germany) according to the manufacturer's protocol. RNA yields were in the range of 0.5 to 100 ng/μL. Primers specific for the *L* genes of FIWIV, KANDV, LOEBV and OBLV were designed based on available GenBank sequences and used for RT‐PCR on RNA extracts (Table S1). For the initial RT‐PCR screening, organs of each animal were examined in pools. Subsequently, individual organs of positive animals were tested separately, using ~350 ng of RNA extract per reaction. Briefly for RT‐PCR, we used the Sybr Green GoTaq 1‐step RT qPCR kit (Promega, USA) with primer concentrations of 200 pM and the following temperature settings: 15 min, 42°C; 10 min, 95°C; 40× (10 s, 95°C; 20 s, 52°C; 30 s, 72°C). A tissue RNA extract of the initially described filovirus positive RNA pool (Hierweger et al. 2021) served as positive control. Amplicons were analysed by agarose gel electrophoresis (1.5% [w/v]). Hight‐throughput sequencing (HTS) was undertaken on selected RNA extracts as described previously at a read depth of ~200 M paired‐end reads (300 cycles) on an Illumina NovaSeq machine (Hierweger et al. 2021). Reads were then aligned to filovirus genome sequences using Geneious prime (Graphpad, USA, version 2025.0.3).

## 3 Results

The analysed perch had an average weight of 125 g, and an average length of 20 cm (Table S2). The average Fultan's condition index *F* was 1.5 (range 1.2–1.8), indicating a good nutritional status (Fishbase 2025). All 32 perch were adult females and showed neither external nor internal organ alterations.

Ten out of 32 examined perch (31.3%) were RT‐PCR positive for at least one of the four tested filoviruses (Table 2; Figures S1–S4). Nine fish were positive for OBLV and KNDV, and one fish was positive for OBLV, KNDV and LOEBV (Table 2). The viruses were detected in the spleen, kidney and pancreas. Only OBLV was also detected in the liver. Gills and brains were always negative (Table 2).

**TABLE 2:** Summary of all animals and organs that were tested positive for Loetschberg virus (LOEBV), Oberland virus (OBLV) and Kander virus (KNDV).

Fish ID	RT‐PCR result per organ					
	Brain	Liver	Spleen	Kidney	Gills	Pancreas
2	nd	nd	OBLV, KNDV	OBLV, KNDV	nd	OBLV, KNDV
4	nd	nd	OBLV, KNDV	OBLV	nd	nd
7	nd	nd	OBLV, KNDV	nd	nd	OBLV
9	nd	OBLV	OBLV, KNDV	nd	nd	KNDV
12	nd	OBLV	nd	KNDV	nd	KNDV
19	nd	OBLV	OBLV	OBLV, KNDV	nd	OBLV
24	nd	nd	KNDV	nd	nd	KNDV
26	nd	nd	OBLV, KNDV	OBLV, KNDV	nd	KNDV
27	nd	nd	OBLV	nd	nd	nd
28	nd	nd	LOEBV, OBLV, KNDV	LOEBV	nd	LOEBV, KNDV

Abbreviation: nd, not detected.

To confirm the RT‐PCR results and to obtain viral sequence data, we conducted HTS on the spleen samples of animal 28 (positive for LOEBV, KNDV and OBLV) and animal 26 (positive for KNDV and OBLV). In both samples, the presence of the different viruses was confirmed by HTS and alignment of HTS reads to the respective genomic sequences (Table 3).

**TABLE 3:** Confirmation of filoviruses in spleen samples of two RT‐PCR positive European perch.

Fish	Total reads	Reference	Mapped reads	Genome coverage (%)	Mean read depth	Pairwise identity (%)
26	257 M	KNDV	1091	87	7.6×	99.0
		OBLV	985	83	6.5×	98.7
28	267 M	KNDV	2441	91	17×	99.0
		OBLV	2887	85	19×	98.7
		LOEBV	6827	100	47×	98.9

*Note:* Data were generated by mapping high‐throughput sequencing reads to perch filovirus genomic sequences.

Abbreviations: KNDV, Kander virus (GenBank accession NC_076916); LOEBV, Loetschberg virus (OQ186623); OBLV, Oberland virus (NC_076735).

## 4 Discussion

Our results corroborate the findings of our previous report on KNDV, OBLV and LOEBV infections of European perch. Noteworthy, the perch sampled in the first batch in 2021 were juvenile, whereas the perch in the second batch were adult animals, demonstrating that filovirus infections are not limited to juvenile perch but can also be found in adult animals. This detection of the KNDV, OBLV and/or LOEBV in 31% of the second animal batch suggests persistence of these filoviruses in the same European perch population over at least 6 years. The identity of these filoviruses was confirmed by HTS. While the viral sequences in the second batch were highly similar to the sequences of the first batch, they were not fully identical (Table 3). These differences may be due to sequencing errors or the ongoing evolutionary adaptation of the virus to the host.

We detected KNDV, OBLV and LOEBV in kidney, spleen and pancreas samples, and most perch were infected by more than one filovirus (Table 2). Additionally, OBLV was detected in the liver samples of three animals. These results overcome the limitation of our previous study using pooled organs and contribute to knowledge of filovirus target tissues in fish, which is relevant information for diagnostic or research sample selection in future studies. Noteworthy, the target tissues we found in the perch are different from those of previously described Huangjiao and Xilang filoviruses (Shi et al. 2018) in captured sea fish in which both viruses were only found in gills but not in gut and liver samples. Since gills are organs permanently exposed to the environment, it was not known whether the filoviruses had infected the fish or rather were a contamination from external sources. Our findings indicate that KNDV, OBLV and LOEBV indeed infect the European perch, and the organ distribution suggests that this infection is most probably systemic.

Several methodological limitations of our study need consideration. An RT‐qPCR protocol for quantification of filovirus loads in the perch organs still needs to be established. Attempts to do so were unsuccessful because we found no robust linear correlation between the RNA concentration and the RT‐qPCR cq value. Furthermore, due to limitations in tissue quality, the target cells of filoviruses in perch using in situ hybridisation (ISH) remain to be assessed. In mammals, filoviruses typically target macrophages and dendritic cells (Warfield et al. 2009), whereas the exact target cell types in fish are not known yet. Our preliminary ISH results suggest that filoviruses are only present in scattered cells throughout the tissue (data not shown), which remains to be investigated further.

All fish were within the range of a normal body condition. We found no pathological lesions in the organs and, overall, no evidence of clinical disease associated with filoviral infection was detected in the fish, supporting that infections with KNDV, LOEBV and OBLV are non‐pathogenic and asymptomatic.

In conclusion, we detected persisting filovirus infection within a perch population. We identified kidney, spleen, pancreas, and liver as target organs within the fish and did not find any evidence for clinical disease associated with the filovirus infections. These results highlight the potential of perch as a valuable model to understand basic filovirus biology. Future studies now need to concentrate on virus isolation in host cells and experimental animal inoculations to further strengthen this model for fundamental research.

## Author Contributions

**Johanna Perschthaler:** inbestigation. methodology, data curation and writing. **Nicole Wildi:** conceptualization, methodology, investigation and data curation. **Lena K. Matthiss:** methodology, investigation and data curation. **Torsten Seuberlich:** conceptualization, supervision, valdiation, writing‐ review and editing, funding aquisation. **Heike Schmidt‐Posthaus:** conceptualization, supervision, valdiation, funding aquisation. writing‐ review and editing

## Funding

This study was funded by institutional resources of the Institute for Fish and Wildlife Health and the Division of Neurological Sciences of the Vetsuisse Faculty, University of Bern, Switzerland.

## Conflicts of Interest

The authors declare no conflicts of interest.

## Supporting information


**Data S1:** jfd70105‐sup‐0001‐DataS1.docx.

## Data Availability

All data are available in the Supporting Information.
