# Supplementary material for: Persistence and Organ Tropism of Filoviruses in Farmed European Perch (Perca fluviatilis)
Source: J Fish Dis. 2025 Dec 6;49(5):e70105. doi: 10.1111/jfd.70105 (PMC13051327; doi:10.1111/jfd.70105)
Supplement: Supplementary file 1 — Data S1: jfd70105‐sup‐0001‐DataS1.docx. [file JFD-49-e70105-s001.docx]

**Supplementary information**

**Table S1:** Summary of necropsy findings of perch including sex, weight, length, and Fulton’s condition index F.

| **Fish ID** | **Sex** | **Weight (g)** | **Length (cm)** | **Condition index F** |
| --- | --- | --- | --- | --- |
| 1 | Female | 117.8 | 19.8 | 1.52 |
| 2 | Female | 66.9 | 17 | 1.36 |
| 3 | Female | 119.2 | 20.2 | 1.45 |
| 4 | Female | 76.2 | 18.5 | 1.2 |
| 5 | Female | 121.8 | 19.9 | 1.55 |
| 6 | Female | 108.8 | 20 | 1.36 |
| 7 | Female | 100.7 | 20 | 1.26 |
| 8 | Female | 136.1 | 21 | 1.47 |
| 9 | Female | 119.6 | 19.6 | 1.59 |
| 10 | Female | 133.8 | 21 | 1.44 |
| 11 | Female | 152.1 | 20.2 | 1.85 |
| 12 | Female | 101 | 19.2 | 1.43 |
| 13 | Female | 122.4 | 20.6 | 1.4 |
| 14 | Female | 104.4 | 19.1 | 1.5 |
| 15 | Female | 77.9 | 17.2 | 1.53 |
| 16 | Female | 99.5 | 18.5 | 1.57 |
| 17 | Female | 78.1 | 17.1 | 1.56 |
| 18 | Female | 81.3 | 18.5 | 1.28 |
| 19 | Female | 118.4 | 20.1 | 1.46 |
| 20 | Female | 127.5 | 20.4 | 1.5 |
| 21 | Female | 111.1 | 19 | 1.62 |
| 22 | Female | 95.1 | 19.1 | 1.36 |
| 23 | Female | 126.8 | 20.2 | 1.54 |
| 24 | Female | 96.3 | 18.5 | 1.52 |
| 25 | Female | 187.3 | 21.2 | 1.97 |
| 26 | Female | 148.2 | 21.1 | 1.58 |
| 27 | Female | 140.9 | 22.1 | 1.31 |
| 28 | Female | 106.1 | 19.5 | 1.43 |
| 29 | Female | 120.5 | 20.4 | 1.42 |
| 30 | Female | 142.8 | 20.6 | 1.63 |
| 31 | Female | 158 | 21.5 | 1.59 |
| 32 | Female | 403.8 | 28.5 | 1.74 |
| Average |  | 125 | 20 | 1.5 |

**Table S2:** Primer sequences and amplified fragment sizes in for RT PCR of Loetschberg virus (LOEBV), Oberland virus (OBLV), FIWI virus (FIWIV), and Kander virus (KNDV). Primers target the L gene of these viruses.

| **Primer** | **Sequence (5’-3’)** | **Amplicon size** |
| --- | --- | --- |
| LOEBV forward | ATGTGGCTTTGGCGTTTTCC | 179 bp |
| LOEBV reverse | TTCATGGCGGACAAGGTACC |  |
| OBLV forward | TGGTCCGCAAATTCGCTACT | 215 bp |
| OBLV reverse | AGGCTCCCTACCTTCATCGT |  |
| FIWIV forward | CTCAAGTGCAGTCTGGACGT | 205 bp |
| FIWIV reverse | TAGACTCGACCACCCTCAGG |  |
| KNDV forward | AAACCTTTGTGGCAGGGTCA | 181 bp |
| KNDV reverse | CCTCTCCCCACAGATCTGGA |  |

*
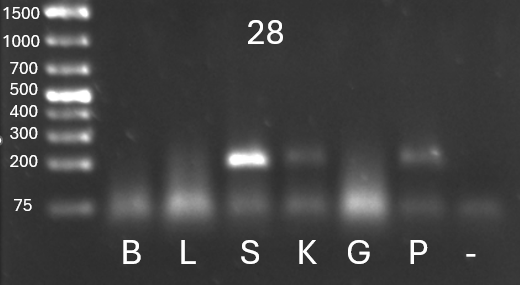
*

**Figure 1:** Organs of perch 28 showing positive RT-PCR results for Loetschberg virus (LOEBV), B = brain, L = liver, S = spleen, K = kidney, G = gills, P = pancreas, - = negative control. Expected amplicon size was 179 bp.

*
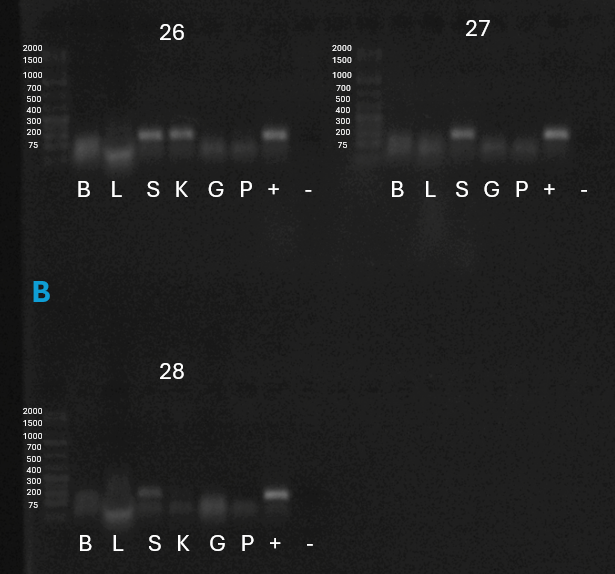

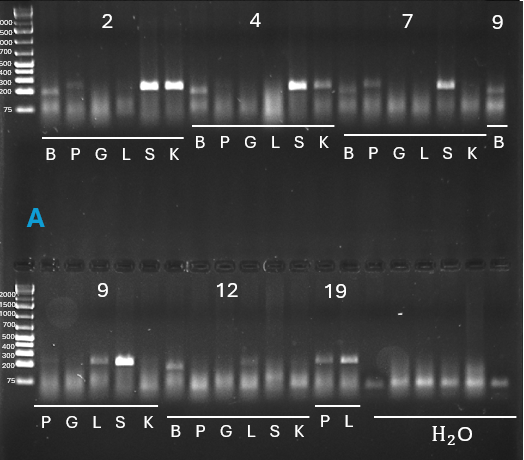
*

**Figure 2:** A: Organs of perch 2, 4, 7, 9, 12, and 19 showing positive RT-PCR results for Oberland virus (OBLV); B: Organs of perch 26, 27, and 28 showing positive RT PCR results for OBLV; B = brain, L = liver, S = spleen, K = kidney, G = gills, P = pancreas, + = positive control, - = negative control. Expected amplicon size was 215 bp.


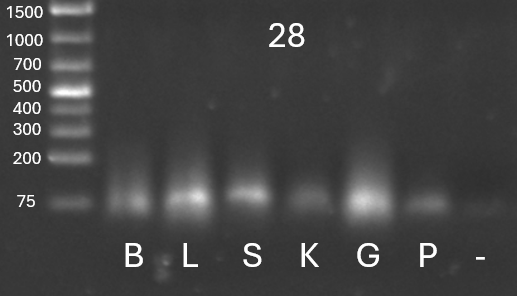


**Figure 3: A:** Organs of perch 28 showing negative RT PCR results for FIWI virus (FIWIV); B = brain, L = liver, S = spleen, K = kidney, G = gills, P = pancreas, - = negative control. Expected amplicon size was 205 bp.


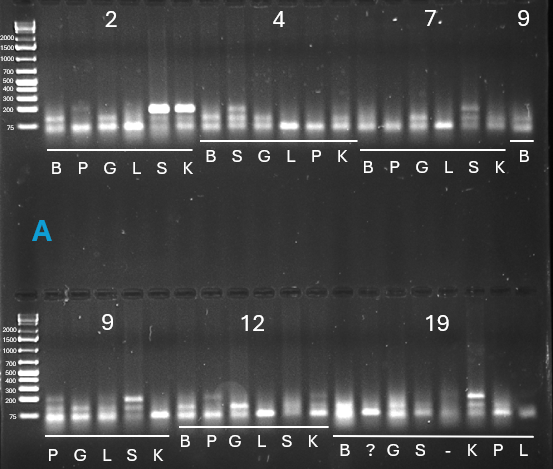
*
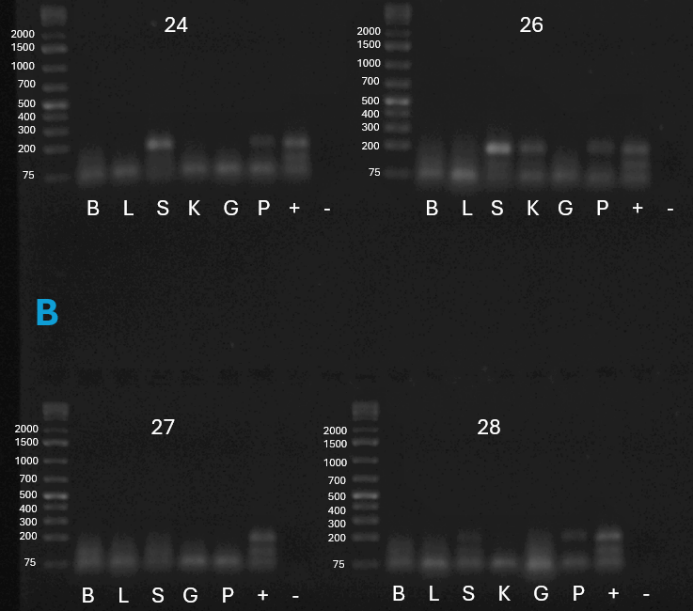
*

**Figure 4:** A: Organs of pools of animals 2, 4, 7, 9, 12, and 19 showing positive results for Kander virus (KNDV), B: Organs of pools of animals 24, 26, 27, and 28 showing positive results for KNDV; B = brain, L = liver, S = spleen, K = kidney, G = gills, P = pancreas, + = positive control, - = negative control. Expected amplicon size was 181 bp.
